# Comparison of outcomes after carotid endarterectomy between type 2 diabetic and non-diabetic patients with significant carotid stenosis

**DOI:** 10.1186/s12933-019-0848-7

**Published:** 2019-03-25

**Authors:** Min-Jae Jeong, Hyunwook Kwon, Chang Hee Jung, Sun U. Kwon, Min-Ju Kim, Youngjin Han, Tae-Won Kwon, Yong-Pil Cho

**Affiliations:** 1 Department of Surgery, University of Ulsan College of Medicine and Asan Medical Center, Asanbyeongwon-gil 86, Songpa-gu, Seoul, 05505 Republic of Korea; 2 Department of Internal Medicine, University of Ulsan College of Medicine and Asan Medical Center, Asanbyeongwon-gil 86, Songpa-gu, Seoul, 05505 Republic of Korea; 3 Department of Neurology, University of Ulsan College of Medicine and Asan Medical Center, Asanbyeongwon-gil 86, Songpa-gu, Seoul, 05505 Republic of Korea; 4 Department of Clinical Epidemiology and Biostatistics, University of Ulsan College of Medicine and Asan Medical Center, Asanbyeongwon-gil 86, Songpa-gu, Seoul, 05505 Republic of Korea

**Keywords:** Carotid artery stenosis, Carotid endarterectomy, Diabetes mellitus, Outcomes

## Abstract

**Background:**

We aimed to compare early and late outcomes after carotid endarterectomy (CEA) between Korean type 2 diabetic and non-diabetic patients and to investigate the impact of diabetes on the overall incidence of cardiovascular events after CEA.

**Methods:**

We retrospectively analyzed 675 CEAs, which were performed on 613 patients with significant carotid stenosis between January 2007 and December 2014. The CEAs were divided into a type 2 diabetes mellitus (DM) group (n = 265, 39.3%) and a non-DM group (n = 410, 60.7%). The study outcomes included the incidence of major adverse events (MAEs), defined as fatal or nonfatal stroke or myocardial infarction or all-cause mortality, during the perioperative period and within 4 years after CEA.

**Results:**

Patients in the DM and non-DM groups did not differ significantly in the incidence of MAEs or any of the individual MAE manifestations during the perioperative period. However, within 4 years after CEA, the difference in the MAE incidence was significantly greater in the DM group (P = 0.040). Analysis of the individual MAE manifestations indicated a significantly higher risk of stroke in the DM group (P = 0.006). Multivariate analysis indicated that diabetes was not associated with MAEs or individual MAE manifestations during the perioperative period, whereas within 4 years after CEA, diabetes was an independent risk factor for MAEs overall (hazard ratio [HR], 1.62; 95% confidence interval [CI] 1.06–2.48; P = 0.026) and stroke (HR, 2.55; 95% CI 1.20–5.41; P = 0.015) in particular.

**Conclusions:**

Diabetic patients were not at greater risk of perioperative MAEs after CEA; however, the risk of late MAE occurrence was significantly greater in these patients. Within 4 years after CEA, DM was an independent risk factor for the occurrence of MAEs overall and stroke in particular.

**Electronic supplementary material:**

The online version of this article (10.1186/s12933-019-0848-7) contains supplementary material, which is available to authorized users.

## Background

It is well established that type 2 diabetes mellitus (DM) is a progressive disease that contributes significantly to the deterioration of atherosclerotic changes of small- to medium-sized arteries [1–3]. Although previous studies have assessed the association between diabetes and higher operative risk during open vascular procedures, there are conflicting results across multiple studies [4–6]. Furthermore, despite the decline of the incidence of diabetes-related morbidity and mortality between 1990 and 2010, owing to improvements in medical treatment [7], overall cardiovascular morbidity and mortality rates are still substantially higher among diabetic patients, particularly among aging patients (who continue to increase in numbers) [8, 9]. Carotid endarterectomy (CEA) is a well-known durable procedure for preventing recurrent neurological symptoms and strokes among patients with significant carotid artery stenosis [10–12]. However, the long-term benefits of stroke prevention after CEA for patients with DM have been subject to debate, with varying conclusions across different studies [6, 8, 9, 13–15].

The prevalence of type 2 DM has been increasing throughout Asia due to the recent Westernization of dietary habits [16]. There may be ethnic disparities in the incidence of diabetes-related cardiovascular morbidity and mortality in the subgroup of DM patients receiving CEA. We aimed to compare early and late outcomes after CEA between Korean type 2 diabetic and non-diabetic patients and to investigate the impact of DM on the overall incidence of cardiovascular events after CEA.

## Methods

### Study design and patient population

In this single-center, retrospective, observational study, we analyzed data extracted from the medical records of patients who underwent CEA. The study protocol was approved by our hospital’s institutional review board, which waived the requirement for informed patient consent given the retrospective nature of the study.

We screened the records of 717 consecutive patients who underwent a total of 789 CEAs at our hospital between January 1, 2007, and December 31, 2014. Among these, 114 CEAs in 104 patients were followed-up at our tertiary medical center within a specified period (less than 1 year); subsequently, once stability had been established, they were followed up at other hospitals and were not included in the analysis of this study. We finally included 675 CEAs (85.6%) conducted on 613 patients who were stratified into two groups: a DM group and a non-DM group. All CEAs had been performed to relieve significant carotid bifurcation stenosis, as defined by velocity criteria and the criteria established by the North American Symptomatic Carotid Endarterectomy Trial (NASCET), as previously published [1, 2, 17–19]: 50–99% luminal narrowing in patients with symptomatic carotid stenosis and ≥ 70% in those with asymptomatic carotid stenosis. Patients who had transient ischemic attacks, amaurosis fugax, or non-disabling stroke ipsilateral to the significant carotid stenosis within the previous 6 months were considered to be symptomatic [20, 21]. When there was a discrepancy between the degree of carotid stenosis determined using velocity criteria and that determined using NASCET luminal narrowing criteria, the estimation of carotid stenosis was based primarily on the velocity criteria [17]. For patients with bilateral significant carotid stenosis, the most symptomatic or higher-grade carotid stenosis was treated first, as previously detailed [20, 22].

Demographics, risk factors, clinical characteristics, and other data—including 30-day and 4-year outcomes—were recorded for all consecutive CEAs in an Excel (Microsoft Corp., Redmond, WA, USA) database and retrospectively analyzed.

### CEA procedure and definitions

The CEA procedure used has been previously detailed [20, 21]. CEAs were performed either under general anesthesia with routine carotid shunting or under regional anesthesia with selective shunting. During the early period of our study, CEA was preferably performed under regional anesthesia, and general anesthesia was selectively used for patients who did not tolerate regional anesthesia, whereas, in the late period, we changed the anesthetic technique for general anesthesia with routine shunting. The preferred option for CEA is an endarterectomy with patch angioplasty in the standard fashion, as previously described [20, 21]. Postoperatively, all patients were given dual antiplatelet therapy with a statin in combination with stringent control of blood pressure and close observation in an intensive care unit for at least 24 h. All patients were followed up both clinically and by magnetic resonance imaging with angiography before discharge.

The diagnosis of type 2 DM was based on plasma glucose criteria, defined by the fulfillment of at least two out of the three the plasma glucose criteria, as published previously [23]. In the absence of unequivocal hyperglycemia, results were confirmed through repeat testing [1]. Patients who reported taking antidiabetic medications (oral hypoglycemic agents or insulin) on a self-administered questionnaire were deemed to have type 2 DM. The duration of DM was estimated as the difference between the age at CEA and the age at DM onset.

Demographics and risk factors were defined as previously published [1]. Body mass index was defined as weight (kg) divided by height squared (m^2^). Hypertension was defined as the use of antihypertensive medications, a systolic blood pressure > 140 mmHg, or diastolic blood pressure > 90 mmHg (mean of two readings taken by the examining physician). Dyslipidemia was defined as the use of lipid-lowering medications, a fasting total serum cholesterol level > 200 mg/dL, a low-density lipoprotein cholesterol level > 120 mg/dL, a high-density lipoprotein cholesterol level < 40 mg/dL, or a triglyceride level > 150 mg/dL. Coronary artery disease (CAD) was defined as ischemic heart disease, as previously published [1]. The diagnosis of subclinical CAD was based on preoperative radionuclide adenosine stress myocardial perfusion imaging and additional coronary computed tomography angiography or coronary angiography in patients without a history of CAD [18, 24, 25]. Chronic kidney disease (CKD) was defined as an estimated glomerular filtration rate < 60 mL min^−1^ × 1.73 m^−2^, which was assessed by using the Modification of Diet in Renal Disease formula [26]. Peripheral arterial occlusive disease (PAOD) was defined when a patient had a previous history of radiological or surgical intervention for PAOD or an ankle–brachial index ≤ 0.9, as measured with Doppler ultrasound (Vasoguard QVL P84, Viasys Healthcare UK Ltd., Old Woking, UK) [27]. Severe contralateral extracranial carotid stenosis or occlusion (SCSO) was defined as ≥ 70% luminal narrowing of the contralateral extracranial carotid stenosis or occlusion [28].

### Outcomes of interest and follow-up

The study outcomes of interest were the occurrence of major adverse events (MAEs), defined as fatal or nonfatal stroke or myocardial infarction (MI) and all-cause mortality during the perioperative period (within 30 days after CEA) and within 4 years after CEA [29]. Only the first event of each outcome was included in the analysis of the MAEs incidence.

Strokes were defined as the occurrence of an acute neurological event with focal symptoms and signs lasting for at least 24 h, which were consistent with focal cerebral ischemia, and were categorized as major or minor [1]. We included only ischemic strokes in the analysis. MI was defined as any increase in creatine kinase–myocardial band or cardiac troponin I above the upper limit of the reference range, with either chest pain, symptoms consistent with ischemia, or electrocardiographic evidence of ischemia (i.e., new ST segment depression or elevation, or > 1 mm elevation in two or more contiguous leads) during follow-up [1]. Following CEA, restenosis was defined as the development of ≥ 70% stenosis, diagnosed based on Doppler ultrasound criteria of the luminal narrowing with a peak systolic velocity threshold of ≥ 274 cm/s [30].

Follow-up visits, with laboratory evaluations and carotid duplex ultrasonography (iU22, Philips Ultrasound, Bothell, WA, USA) as well as independent neurological examinations, were scheduled at 6 and 12 postoperative months, and annually thereafter. Follow-up cardiac enzyme levels and 12-lead electrocardiograms were performed depending on individual atherosclerosis risk factors. Once stability had been established over 3 years, surveillance was performed at longer intervals of about 2 years. For antidiabetic medications and glycemic control, a substantial proportion of patients received medication adjustments and assessments to evaluate glycemic or metabolic control with follow-up at other hospitals once stability had been established at our diabetes center.

### Statistical analysis

Categorical variables are reported as frequencies or percentages, and continuous variables as means and standard deviations. Categorical variables were compared by using the Chi square test or Fisher’s exact test, as appropriate, whereas continuous variables were compared using Student’s t-test. Univariate and multivariate logistic regression analyses were used to identify the association between clinical variables and perioperative outcomes (within 30 days after CEA), and odds ratios (ORs) with 95% confidence intervals (CIs) are reported. The cumulative probabilities of long-term event-free rates in terms of MAE-free, stroke-free, and overall survival rates in the two groups were estimated using Kaplan–Meier analysis and compared with estimations calculated using the log-rank test. Univariate and multivariate analyses of the association between clinical variables and long-term outcomes (within 4 years after CEA) were conducted with Cox proportional hazard regression modeling, using the event of interest and the period from CEA to the date of the event or last follow-up as the outcome. Univariate Cox proportional hazard regression models were fitted to calculate hazard ratios (HRs) with 95% CIs to estimate the associations between clinical variables and outcomes. Variables with a P-value of < 0.1 on univariate analysis were included in the multivariate analysis using the backward elimination method. A P-value < 0.05 was considered statistically significant. Statistical analyses were performed using SPSS version 21.0 (IBM Corp., Armonk, NY, USA).

## Results

The study cohort consisted of 675 CEAs in 613 patients who underwent CEA at our hospital. The DM group consisted of 265 CEAs (39.3%), and the non-DM group consisted of 410 CEAs (60.7%). The baseline and clinical characteristics of the patients according to DM status are presented in Table 1. In the DM group, the mean diabetes duration was 11 years, and the proportion of insulin use was 17.7% (47/265). Regarding atherosclerotic risk factors and comorbidities, the DM group had a higher prevalence of hypertension (P = 0.007), CAD (P < 0.001), CKD (P = 0.001), and PAOD (P = 0.003) than the non-DM group. There were no significant differences in the degree of carotid stenosis, SCSO and the proportion of symptomatic stenosis, or the anesthetic and reconstruction techniques of CEA between the two groups. Regarding antidiabetic medications and glycemic control, 63.8% of patients (169/265) in the DM group received medication adjustments and assessments to evaluate glycemic or metabolic control with follow-up at other hospitals.

**Table 1:** Baseline and clinical characteristics of the study population stratified according to diabetes status

	Total	DM	Non-DM	P-value
No. of CEAs	675	265 (39.3)	410 (60.7)	
Mean age (years)	68.5 ± 7.7	68.1 ± 7.3	68.8 ± 7.9	0.222
Male sex	590 (87.4)	228 (86.0)	362 (88.3)	0.389
BMI (kg/m^2^)	24.1 ± 2.9	24.2 ± 3.0	24.0 ± 2.8	0.669
Diabetes duration (years)	NA	11.3 ± 9.8	NA	NA
Hypoglycemic medications				
Insulin^a^	NA	47 (17.7)	NA	NA
Metformin^a^	NA	185 (69.8)	NA	NA
Risk factor				
Smoking	450 (66.7)	182 (68.7)	268 (65.4)	0.373
Hypertension	513 (76.0)	216 (81.5)	297 (72.4)	0.007
Dyslipidemia^b^	466 (69.0)	192 (72.5)	274 (66.8)	0.123
Comorbidities				
CAD	131 (19.4)	72 (27.2)	59 (14.4)	< 0.001
Subclinical CAD	23 (3.3)	10 (3.8)	13 (3.2)	0.673
CKD	112 (16.6)	59 (22.3)	53 (12.9)	0.001
PAOD	45 (6.7)	27 (10.2)	18 (4.4)	0.003
Carotid stenosis				
Degree of stenosis (%)	76.2 ± 9.5	76.0 ± 8.6	76.3 ± 10.0	0.705
SCSO	72 (10.7)	29 (10.9)	43 (10.5)	0.851
Symptomatic stenosis	324 (48.0)	116 (43.8)	208 (50.7)	0.077
CEA				
General anesthesia	405 (60.0)	155 (58.5)	250 (61.0)	0.520
Use of shunt	421 (62.4)	168 (63.4)	253 (61.7)	0.658
Reconstruction				0.897
Patch angioplasty	653 (96.7)	257 (97.0)	396 (96.6)	
Primary closure	10 (1.5)	3 (1.1)	7 (1.7)	
Others	12 (1.8)	5 (1.9)	7 (1.7)	

Continuous data are presented as mean ± standard deviation; categorical data are given as number (%)

*BMI* body mass index, *CAD* coronary artery disease, *CEA* carotid endarterectomy, *CKD* chronic kidney disease, *DM* diabetes mellitus, *NA* not applicable, *PAOD* peripheral arterial occlusive disease, *SCSO* severe contralateral extracranial carotid stenosis or occlusion

^a^Hypoglycemic medications at the time of CEA

^b^All patients received statins prior to CEA

**Fig. 1:**
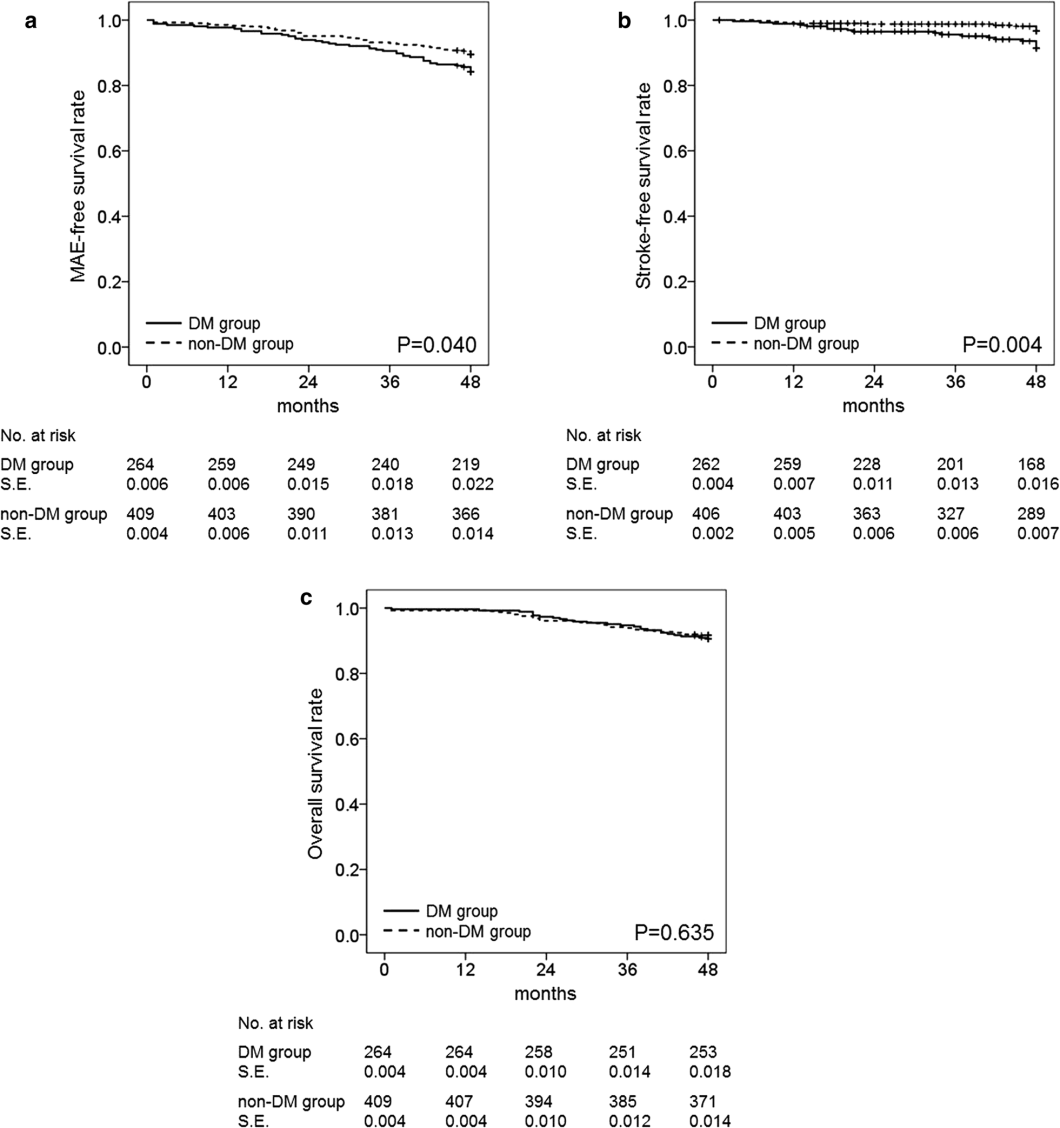
Four-year Kaplan–Meier analyses of the cumulative event-free rates. **a** MAE-free, **b** stroke-free, and **c** overall survival rates among patients in the DM and non-DM groups who underwent carotid endarterectomy. *DM* diabetes mellitus, *MAE* major adverse event; *S.E.* standard error

Patients in the DM and non-DM groups did not differ significantly in terms of overall incidence of MAE occurrence (2.6% versus 2.0%, respectively; P = 0.552) or incidence of any of the individual MAE manifestations during the perioperative period. However, within 4 years after CEA, the MAE incidence was found to be 15.8% in the DM group and 10.5% in the non-DM group (P = 0.040) (Table 2). Analysis of the individual MAE manifestations indicated a significantly higher risk of stroke in the DM group (P = 0.006), whereas there were no significant differences between the two groups in the risks of MI and all-cause mortality. The risk of minor stroke was significantly higher in the DM group (6.4% *versus* 1.7%; P = 0.001); however, no significant difference was noted in the incidence of major stroke (0.8% versus 1.0%; P = 0.999) between the two groups. On Kaplan–Meier survival analysis, although there was a similar overall survival rate (P = 0.635) between the two groups, the DM group had lower MAE-free (P = 0.040) and stroke-free (P = 0.004) survival rates compared with the non-DM group (Fig. 1). The MAE-free, stroke-free, and overall survival rates at 4 years in the DM and non-DM groups were 85.7% and 90.5%, 93.6% and 98.1%, and 90.6% and 91.7%, respectively. During the study period, restenosis occurred after 12 CEAs (1.8%): 5 CEAs (1.9%) in the DM group and 7 CEAs (1.7%) in the non-DM group (P = 0.999). There was no restenosis-related stroke.

**Table 2:** Major adverse events (MAEs) and the individual MAE components among patients who have undergone carotid endarterectomy, according to diabetes status

	Within 30-day outcomes after CEA				Within 4-year outcomes after CEA^a^			
	Total (n = 675)	DM (n = 265)	Non-DM (n = 410)	P-value	Total (n = 675)	DM (n = 265)	Non-DM (n = 410)	P-value
MAE	15 (2.2)	7 (2.6)	8 (2.0)	0.552	85 (12.6)	42 (15.8)	43 (10.5)	0.040
Any stroke	9 (1.3)	4 (1.5)	5 (1.2)	0.743	30 (4.4)	19 (7.2)	11 (2.7)	0.006
Major	1 (0.1)	0	1 (0.2)	0.999	6 (0.9)	2 (0.8)	4 (1.0)	0.999
Minor	8 (1.2)	4 (1.5)	4 (1.0)	0.718	24 (3.6)	17 (6.4)	7 (1.7)	0.001
MI	2 (0.3)	2 (0.8)	0	0.154	2 (0.3)	2 (0.8)	0	0.154
Death	4 (0.6)	1 (0.4)	3 (0.7)	0.999	59 (8.7)	25 (9.4)	34 (8.3)	0.608

Values in parentheses are percentages

*CEA* carotid endarterectomy, *DM* diabetes mellitus, *MI* myocardial infarction

Any stroke, MI, or death

^a^Including the occurrence of MAEs during the perioperative period

After adjusting for confounding variables, dyslipidemia had a protective effect on perioperative MAE occurrence (OR, 0.30; 95% CI 0.10–0.87; P = 0.027), whereas CAD increased the risk of perioperative MAE 3.74-fold (95% CI 1.25–11.2; P = 0.018) (Table 3). Multivariate analysis indicated that dyslipidemia (OR, 0.16; 95% CI 0.04–0.67; P = 0.012) was an independent determinant of decreased perioperative stroke risk and that CAD (OR, 5.22; 95% CI 1.30–20.90; P = 0.020) was an independent determinant of increased perioperative stroke risk (Additional file 1: Table S1). For the incidence of perioperative MI and all-cause mortality, univariate analysis identified no statistically significant associated factor, which precluded the execution of multivariate analysis (data not shown).

**Table 3:** Factors associated with the occurrence of 30-day major adverse events

	Univariate analysis		Multivariate analysis	
	OR (95% CI)	P-value	OR (95% CI)	P-value
Age	0.99 (0.93–1.06)	0.823	NA	NA
Female sex	0.49 (0.06–3.77)	0.493	0.62 (0.08–4.93)	0.653
BMI	1.13 (0.95–1.35)	0.155	NA	NA
Smoking	1.38 (0.44–4.40)	0.581	NA	NA
Diabetes	1.36 (0.49–3.81)	0.554	NA	NA
Insulin use	0.77 (0.90–6.53)	0.809	NA	NA
Hypertension	4.52 (0.59–34.6)	0.147	NA	NA
Dyslipidemia	0.38 (0.14–1.07)	0.067	0.30 (0.10–0.87)	0.027
CAD	2.85 (1.00–8.16)	0.051	3.74 (1.25–11.20)	0.018
Subclinical CAD	NA	NA	NA	NA
CKD	2.58 (0.87–7.71)	0.089	2.44 (0.80–7.44)	0.118
PAOD	1.00 (0.13–7.78)	0.999	0.80 (0.10–6.51)	0.836
Degree of stenosis	0.99 (0.94–1.04)	0.685	NA	NA
SCSO	2.14 (0.59–7.78)	0.247	NA	NA
Symptomatic stenosis	0.95 (0.34–2.64)	0.917	NA	NA

*BMI* body mass index, *CAD* coronary artery disease, *CI* confidence interval, *CKD* chronic kidney disease, *OR* odds ratio, *NA* not applicable, *PAOD* peripheral arterial occlusive disease, *SCSO* severe contralateral extracranial carotid stenosis or occlusion

After adjustment for potential confounding variables, multivariate analysis of the association between clinical variables and long-term incidence of MAEs indicated that older age (HR, 1.05; 95% CI 1.02–1.08; P = 0.001) and DM (HR, 1.62; 95% CI 1.06–2.48; P = 0.026) were independent risk factors for MAE occurrence (Table 4). According to the analyses of the association between clinical variables and individual MAE manifestations, DM (HR, 2.55; 95% CI 1.20–5.41; P = 0.015) was significantly associated with an increased risk of stroke within 4 years after CEA (Additional file 1: Table S2). Older age (HR, 1.06; 95% CI 1.01–1.12; P = 0.023) and insulin use (HR, 2.34; 95% CI 1.02–5.37; P = 0.045) were independently associated with an increased 4-year any-cause mortality rate (Additional file 1: Table 3). There was no statistically significant factor associated with an increased 4-year MI incidence (data not shown).

**Table 4:** Factors associated with the occurrence of 4-year major adverse events

	Univariate analysis		Multivariate analysis	
	HR (95% CI)	P-value	HR (95% CI)	P-value
Age	1.05 (1.02–1.08)	0.002	1.05 (1.02–1.08)	0.001
Female sex	1.15 (0.63–2.13)	0.646	1.06 (0.57–1.95)	0.865
BMI	0.95 (0.88–1.03)	0.192	NA	NA
Smoking	1.36 (0.85–2.20)	0.204	NA	NA
Diabetes	1.55 (1.02–2.38)	0.042	1.62 (1.06–2.48)	0.026
Insulin use	1.71 (0.86–3.39)	0.129	NA	NA
Hypertension	0.70 (0.44–1.10)	0.123	NA	NA
Dyslipidemia	0.81 (0.52–1.27)	0.356	NA	NA
CAD	1.36 (0.83–2.22)	0.225	NA	NA
Subclinical CAD	0.33 (0.05–2.34)	0.265	NA	NA
CKD	1.60 (0.97–2.65)	0.065	1.27 (0.76–2.15)	0.362
PAOD	0.88 (0.36–2.17)	0.783	0.77 (0.31–1.90)	0.565
Degree of stenosis	1.01 (0.99–1.03)	0.379	NA	NA
SCSO	1.26 (0.67–2.38)	0.472	NA	NA
Symptomatic stenosis	0.83 (0.54–1.28)	0.397	NA	NA

*BMI* body mass index, *CAD* coronary artery disease, *CI* confidence interval, *CKD* chronic kidney disease, *HR* hazard ratio, *NA* not applicable, *PAOD* peripheral arterial occlusive disease, *SCSO* severe contralateral extracranial carotid stenosis or occlusion

## Discussion

### Ethnic differences and optimal management strategy for DM patients with carotid stenosis

Although our study cohort consisted of only Koreans and may not be representative of other ethnic groups, the prevalence of type 2 DM has been increasing throughout Asia, and the speed of this increase is much faster than in Western countries [16]. In Korea, the prevalence of DM increased slightly between 2007 and 2014: 7.7% to 8.0% in the general population and 30.7% to 32.4% in the stroke population [31]. The prevalence of DM among Korean adults is expected to rise to 11.4% by 2030, accelerated by the aging of the population [32]. A large-scale meta-analysis project, the Asia Pacific Cohort Studies Collaboration, showed that the HRs of DM for ischemic stroke and MI are similar between Asian and Western countries [33]; moreover, glucose intolerance and DM are risk factors for stroke and MI in most Asian countries, as they are in Western countries [34–37]. Similar results were reported by the recently published “Stroke Statistics in Korea” project [31]; DM is an important risk factor for stroke. Although stroke mortality is gradually declining, it remains as high as 30 deaths per 100,000 individuals in Korea [31]. The trends of increased DM prevalence, higher risks of DM-related significant carotid stenosis and stroke occurrence highlight the importance of carotid revascularization as a primary or secondary preventive management strategy for this fast-growing, vulnerable population in Korea.

Although carotid artery stenosis patients undergoing CEA or carotid artery stenting (CAS) had similar 30-day readmission rates [38], Columbo et al. [39] recently reported that patients who undergo CEA have a long-term survival advantage over those who undergo CAS in real-world practice, despite no difference in long-term survival in randomized trials. Furthermore, according to a recent multicenter study in Korea, although the risk of major adverse cardiovascular events did not differ significantly within 4 years among Korean CAS and CEA patients, there was a higher risk of stroke with CAS during the periprocedural period [40]. However, the optimal management strategy for DM patients with significant carotid stenosis remains to be defined, because randomized clinical trials have focused on comparing the effectiveness of CEA and CAS for high-risk [41] or standard-risk [29, 42, 43] patients, with minimal specific focus on patients with DM [4, 9]. Moreover, controversy exists about the long-term benefit of stroke prevention after CEA in the diabetic population, with varying conclusions across different Western studies [6, 8, 13–15]. There have been few reports to document the impact of DM on early and late outcomes after CEA in Asian populations.

In our study, we compared the outcomes after CEA between diabetic and non-diabetic patients and found that DM patients are not at greater risk of 30-day MAEs after CEA compared with non-DM patients; however, the 4-year risk MAE occurrence is significantly greater among DM patients. Following the perioperative period, the rate of major stroke was less than 1.0% at 4 years in both DM and non-DM patients. However, the rate of stroke (of any severity) was 7.2% at 4 years in the DM group compared with 2.7% in the non-DM group, confirming that the efficacy of CEA for stroke prevention might be poorer in the long term in the presence of DM. Nevertheless, the inclusion of minor neurologic complications influenced significance for the combined outcome. Furthermore, patients in the DM group had a higher prevalence of atherosclerotic risk factors and DM-related comorbidities than those in the non-DM group. In addition to DM itself, these baseline differences also affected the 4-year MAE incidence differences between the two groups, and the present observations corroborate a report by Adegbala et al. [9], which found that the presence of DM with chronic complications is an important risk factor for poorer outcomes after CEA.

Considering that there are conflicting reports on the impact of DM on the outcomes of patients who have undergone CEA, our findings are also inconsistent with some previously reported results, almost all of which were from studies on Western populations [4, 8, 13, 44]. Although our study sample was small, the discrepancies between our findings and those of previous studies are likely attributable in part to ethnic disparities between Asian and Western populations. There are limited data available derived from studies on Asian populations, and therefore our findings could help inform clinicians about the best treatment options for Asian DM patients with significant carotid stenosis. Further studies of larger cohorts are needed to better understand the impact of DM on clinical outcomes following CEA in Asian populations.

### Impact of risk factors on outcomes after CEA

The deleterious effects of obesity on patient survival in the general population are well known [45]. Among patients with metabolic syndrome, obesity is a confirmed independent risk factor for carotid plaque destabilization, particularly among males aged < 70 years [46]. In our analysis, increased body mass index (BMI) within the normal range was not associated with increased risk of 30-day or 4-year MAE occurrence after CEA. Although the recent Westernization of dietary habits has resulted in increasing mean BMI in most Asian countries, Asian populations remain less obese than Western populations.

As for other atherosclerosis risk factors, dyslipidemia, diagnosed before CEA, has a protective effect on 30-day MAE and stroke occurrence, whereas there is no significant impact of dyslipidemia on the 4-year risks of MAEs or the individual MAE manifestations. Two randomized trials and several cohort studies have demonstrated the effectiveness of a short preoperative statin course to improve the 30-day outcomes of postoperative cardiovascular morbidity and mortality after major vascular surgery [44, 47–51]. Considering that all patients diagnosed with dyslipidemia received statins before CEA in our study population, this potentially explained our observation of a protective effect of dyslipidemia on 30-day MAE and stroke occurrence. This current finding adds support to the previous report by Visser et al. [44], which found that statin use is significantly associated with a decreased risk of MAEs after CEA.

Other data reveal the association between medical management of DM and an increased risk of MAE occurrence among DM patients taking insulin [52–54]. The need for insulin might be associated with more than a sevenfold increased risk of perioperative stroke and death after CEA. This may suggest that a more advanced stage of DM with chronic complications, or a different metabolic status requiring more aggressive glycemic control among DM patients requiring insulin, could lead to higher risk of ischemic events and death [8]. Concerning 4-year any-cause mortality, our data, consistent with previous studies, showed that insulin use was significantly associated with a worse 4-year survival rate. The presence of DM might both increase neointimal hyperplasia and accelerate the growth of new carotid plaques at the site of arterial injury [8], thereby implying an increased risk of restenosis after CEA [14, 55]. However, this hypothesis has remained controversial; our study, as well as others in the literature [5, 56], indicated that DM patients have similar restenosis rates after CEA compared with non-DM patients.

### Study limitations

It is clear that our study has substantial limitations, including its retrospective design and small sample size in a single-center cohort. There was potential for selection and information biases on the part of the physicians or patients, owing to the retrospective study design; hence, the incidence of MAEs may have been underestimated, and the number of excluded patients was considerable. Furthermore, we calculated the DM duration at baseline using patient self-reported age at DM onset, which may have been inaccurate owing to the lag times between disease onset, diagnosis, and self-reporting for study purposes. Additionally, several important factors were not available from our data sources, such as biochemical assessments to evaluate glycemic or metabolic control among DM and non-DM patients. Patient adherence to prescribed DM medications was not supervised, because a substantial proportion of patients were followed up at other hospitals once stability had been established at our DM center. Owing to the lack of data on serial measurements of glycated hemoglobin levels and other DM-related factors, we could not account for differences in glycemic control in this analysis. Furthermore, the study cohort consisted entirely of Korean Asians; therefore, our results may not be generalizable to other ethnic groups. Finally, as with all observational studies, we cannot draw conclusions about causality.

## Conclusions

DM is one of the most common and disabling diseases with a strong cardiovascular burden. Epidemiologic studies have confirmed that DM independently increases the risk of ischemic stroke and stroke-related functional outcomes and mortality. Therefore, it is expected that the benefit of stroke prevention measures, such as CEA, for those with significant carotid stenosis, might be more substantial for DM patients. In our study, we found that DM patients are not at greater risk of perioperative MAE occurrence after CEA compared with non-DM patients; however, the risk of late MAE occurrence was significantly greater among DM patients. DM was an independent risk factor for MAE and stroke occurrence within 4 years after CEA. Prior history of dyslipidemia—meaning preoperative statin use—had a protective effect against perioperative MAE and stroke occurrence, whereas insulin use negatively affected 4-year survival.

## Additional file

**Additional file 1: Table S1.** Factors associated with the occurrence of stroke within 30 days after carotid endarterectomy. **Table S2.** Factors associated with the occurrence of stroke within 4 years after carotid endarterectomy. **Table S3.** Factors associated with mortality within 4 years after carotid endarterectomy.

## Abbreviations

BMI: body mass index
CAS: carotid artery stenting
CEA: carotid endarterectomy
CKD: chronic kidney disease
CI: confidence interval
CAD: coronary artery disease
DM: diabetes mellitus
HR: hazard ratio
MAE: major adverse event
MI: myocardial infarction
NASCET: North American Symptomatic Carotid Endarterectomy Trial
OR: odds ratio
PAOD: peripheral arterial occlusive disease
SCSO: severe contralateral extracranial carotid stenosis or occlusion

## References

[1] Noh M, Kwon H, Jung CH, Kwon SU, Kim MS, Lee WJ (2017). Impact of diabetes duration and degree of carotid artery stenosis on major adverse cardiovascular events: a single-center, retrospective, observational cohort study. Cardiovasc Diabetol.

[2] Kim JJ, Hwang BH, Choi IJ, Choo EH, Lim S, Kim JK (2015). Impact of diabetes duration on the extent and severity of coronary atheroma burden and long-term clinical outcome in asymptomatic type 2 diabetic patients: evaluation by coronary CT angiography. Eur Heart J Cardiovasc Imaging.

[3] Li MF, Zhao CC, Li TT, Tu YF, Lu JX, Zhang R (2016). The coexistence of carotid and lower extremity atherosclerosis further increases cardio-cerebrovascular risk in type 2 diabetes. Cardiovasc Diabetol.

[4] Hussain MA, Bin-Ayeed SA, Saeed OQ, Verma S, Al-Omran M (2016). Impact of diabetes on carotid artery revascularization. J Vasc Surg.

[5] Paraskevas KI, Baker DM, Pompella A, Mikhailidis DP (2008). Does diabetes mellitus play a role in restenosis and patency rates following lower extremity peripheral arterial revascularization? A critical overview. Ann Vasc Surg.

[6] Goodney PP, Likosky DS, Cronenwett JL, Vascular Study Group of Northern New England (2008). Factors associated with stroke or death after carotid endarterectomy in Northern New England. J Vasc Surg.

[7] Preis SR, Hwang SJ, Coady S, Pencina MJ, D’Agostino RB, Savage PJ (2009). Trends in all-cause and cardiovascular disease mortality among women and men with and without diabetes mellitus in the Framingham Heart Study, 1950 to 2005. Circulation.

[8] Parlani G, De Rango P, Cieri E, Verzini F, Giordano G, Simonte G (2012). Diabetes is not a predictor of outcome for carotid revascularization with stenting as it may be for carotid endarterectomy. J Vasc Surg.

[9] Adegbala O, Martin KD, Otuada D, Akinyemiju T (2017). Diabetes mellitus with chronic complications in relation to carotid endarterectomy and carotid artery stenting outcomes. J Stroke Cerebrovasc Dis.

[10] Barnett HJM, Taylor DW, Haynes RB, Sackett DL, Peerless SJ, Ferguson GG, North American Symptomatic Carotid Endarterectomy Trial Collaborators (1991). Beneficial effect of carotid endarterectomy in symptomatic patients with high-grade carotid stenosis. N Engl J Med.

[11] European Carotid Surgery Trialists’ Collaborative Group (1991). MRC European Carotid Surgery Trial: interim results for symptomatic patients with severe (70–99%) or with mild (0–29%) carotid stenosis. Lancet.

[12] Cunningham EJ, Bond R, Mehta Z, Mayberg MR, Warlow CP, Rothwell PM, European Carotid Surgery Trialists’ Collaborative Group (2002). Long-term durability of carotid endarterectomy for symptomatic stenosis and risk factors for late postoperative stroke. Stroke.

[13] Protack CD, Bakken AM, Xu J, Saad WA, Lumsden AB, Davies MG (2009). Metabolic syndrome: a predictor of adverse outcomes after carotid revascularization. J Vasc Surg.

[14] Dorigo W, Pulli R, Pratesi G, Fargion A, Marek J, Innocenti AA (2011). Early and long-term results of carotid endarterectomy in diabetic patients. J Vasc Surg.

[15] Calvillo-King L, Xuan L, Zhang S, Tuhrim S, Halm EA (2010). Predicting risk of perioperative death and stroke after carotid endarterectomy in asymptomatic patients: derivation and validation of a clinical risk score. Stroke.

[16] Ueshima H, Sekikawa A, Miura K, Turin TC, Takashima N, Kita Y (2008). Cardiovascular disease and risk factors in Asia: a selected review. Circulation.

[17] Kwon H, Kim HK, Kwon SU, Lee SW, Kim MJ, Park JW (2018). Risk of major adverse cardiovascular events in subjects with asymptomatic mild carotid artery stenosis. Sci Rep.

[18] Kwon H, Moon DH, Han Y, Lee JY, Kwon SU, Kang DW (2017). Impact of subclinical coronary artery disease on the clinical outcomes of carotid endarterectomy. J Neurosurg.

[19] Ricotta JJ, Aburahma A, Ascher E, Eskandari M, Faries P, Lal BK, Society for Vascular Surgery (2011). Updated society for vascular surgery guidelines for management of extracranial carotid disease. J Vasc Surg.

[20] Kim A, Kwon TW, Han Y, Kwon SU, Kwon H, Noh M (2015). Clinical outcomes of staged bilateral carotid endarterectomy for bilateral carotid artery stenosis. Ann Surg Treat Res.

[21] Kim JH, Cho YP, Kwon TW, Kim H, Kim GE (2012). Ten-year comparative analysis of bovine pericardium and autogenous vein for patch angioplasty in patients undergoing carotid endarterectomy. Ann Vasc Surg.

[22] Marrocco-Trischitta MM, Melissano G, Kahlberg A, Setacci F, Abeni D, Chiesa R (2006). Increased incidence of cerebral clamping ischemia during early contralateral carotid endarterectomy. J Vasc Surg.

[23] American Diabetes Association (2016). 2. Classification and diagnosis of diabetes. Diabetes Care.

[24] Berman DS, Abidov A, Kang X, Hayes SW, Friedman JD, Sciammarella MG (2004). Prognostic validation of a 17-segment score derived from a 20-segment score for myocardial perfusion SPECT interpretation. J Nucl Cardiol.

[25] Kim YH, Ahn JM, Park DW, Song HG, Lee JY, Kim WJ (2012). Impact of ischemia-guided revascularization with myocardial perfusion imaging for patients with multivessel coronary disease. J Am Coll Cardiol.

[26] Levey AS, Coresh J, Greene T, Stevens LA, Zhang YL, Hendriksen S, Chronic Kidney Disease Epidemiology Collaboration (2006). Using standardized serum creatinine values in the modification of diet in renal disease study equation for estimating glomerular filtration rate. Ann Intern Med.

[27] Norgren L, Hiatt WR, Dormandy JA, Nehler MR, Harris KA, Fowkes FG, TASC II Working Group (2007). Inter-society consensus for the management of peripheral arterial disease (TASC II). Eur J Vasc Endovasc Surg.

[28] Patel PB, LaMuraglia GM, Lancaster RT, Clouse WD, Kwolek CJ, Conrad MF (2018). Severe contralateral carotid stenosis or occlusion does not have an impact on risk of ipsilateral stroke after carotid endarterectomy. J Vasc Surg.

[29] Brott TG, Hobson RW, Howard G, Roubin GS, Clark WM, Brooks W, CREST Investigators (2010). Stenting versus endarterectomy for treatment of carotid-artery stenosis. N Engl J Med.

[30] AbuRahma AF, Stone P, Deem S, Dean LS, Keiffer T, Deem E (2009). Proposed duplex velocity criteria for carotid restenosis following carotid endarterectomy with patch closure. J Vasc Surg.

[31] Kim JY, Kang K, Kang J, Koo J, Kim DH, Kim BJ (2019). Executive summary of stroke statistics in Korea 2018: a report from the Epidemiology Research Council of the Korean Stroke Society. J Stroke..

[32] Kim DJ (2011). The epidemiology of diabetes in Korea. Diabetes Metab J.

[33] Woodward M, Zhang X, Barzi F, Pan W, Ueshima H, Rodgers A, Asia Pacific Cohort Studies Collaboration (2003). The effects of diabetes on the risks of major cardiovascular diseases and death in the Asia-Pacific region. Diabetes Care.

[34] NIPPON DATA80 Research Group (2006). Risk assessment chart for death from cardiovascular disease based on a 19-year follow-up study of a Japanese representative population. Circ J.

[35] Cardoso CRL, Salles GC, Leite NC, Salles GF (2019). Prognostic impact of carotid intima-media thickness and carotid plaques on the development of micro- and macrovascular complications in individuals with type 2 diabetes: the Rio de Janeiro type 2 diabetes cohort study. Cardiovasc Diabetol.

[36] Kosiborod M, Gomes MB, Nicolucci A, Pocock S, Rathmann W, Shestakova MV, DISCOVER investigators (2018). Vascular complications in patients with type 2 diabetes: prevalence and associated factors in 38 countries (the DISCOVER study program). Cardiovasc Diabetol.

[37] de Miguel-Yanes JM, Jiménez-García R, Hernández-Barrera V, Méndez-Bailón M, de Miguel-Díez J, Lopez-de-Andrés A (2017). Impact of type 2 diabetes mellitus on in-hospital-mortality after major cardiovascular events in Spain (2002–2014). Cardiovasc Diabetol.

[38] Hintze AJ, Greenleaf EK, Schilling AL, Hollenbeak CS (2019). Thirty-day readmission rates for carotid endarterectomy versus carotid artery stenting. J Surg Res.

[39] Columbo JA, Martinez-Camblor P, MacKenzie TA, Kang R, Trooboff SW, Goodney PP (2019). A comparative analysis of long-term mortality after carotid endarterectomy and carotid stenting. J Vasc Surg.

[40] Lee J, You JH, Oh SH, Shin S, Kim BM, Kim TS (2019). Outcomes of stenting versus endarterectomy for symptomatic extracranial carotid stenosis: a retrospective multicenter study in Korea. Ann Vasc Surg.

[41] Yadav JS, Wholey MH, Kuntz RE, Fayad P, Katzen BT, Mishkel GJ, Stenting and Angioplasty with Protection in Patients at High Risk for Endarterectomy Investigators (2004). Protected carotid-artery stenting versus endarterectomy in high risk patients. N Engl J Med.

[42] Ringleb PA, Allenberg J, Brückmann H, Eckstein HH, Fraedrich G, Hartmann M, SPACE Collaborative Group (2006). 30 day results from the SPACE trial of stent-protected angioplasty versus carotid endarterectomy in symptomatic patients: a randomised non-inferiority trial. Lancet.

[43] Bonati LH, Dobson J, Featherstone RL, Ederle J, van der Worp HB, de Borst GJ, International Carotid Stenting Study investigators (2014). Long-term outcomes after stenting versus endarterectomy for treatment of symptomatic carotid stenosis: the International Carotid Stenting Study (ICSS) randomised trial. Lancet.

[44] Visser L, Wallis de Vries BM, Mulder DJ, Uyttenboogaart M, Veen SV, Zeebregts CJ (2017). The influence of the metabolic syndrome on the short- and long-term outcome after carotid endarterectomy. Angiology.

[45] Whitlock G, Lewington S, Sherliker P, Clarke R, Emberson J, Halsey J, Prospective Studies Collaboration (2009). Body-mass index and cause-specific mortality in 900 000 adults: collaborative analyses of 57 prospective studies. Lancet.

[46] Rovella V, Anemona L, Cardellini M, Scimeca M, Saggini A, Santeusanio G (2018). The role of obesity in carotid plaque instability: interaction with age, gender, and cardiovascular risk factors. Cardiovasc Diabetol.

[47] Durazzo AE, Machado FS, Ikeoka DT, De Bernoche C, Monachini MC, Puech-Leão P (2004). Reduction in cardiovascular events after vascular surgery with atorvastatin: a randomized trial. J Vasc Surg.

[48] Schouten O, Boersma E, Hoeks SE, Benner R, van Urk H, van Sambeek MR, Dutch Echocardiographic Cardiac Risk Evaluation Applying Stress Echocardiography Study Group (2009). Fluvastatin and perioperative events in patients undergoing vascular surgery. N Engl J Med.

[49] Hoeks SE, Op Reimer WJS, Schouten O, Lenzen MJ, van Urk H, Poldermans D (2008). Statin use in the elderly: results from a peripheral vascular survey in The Netherlands. J Vasc Surg.

[50] Feringa HH, Schouten O, Karagiannis SE, Brugts J, Elhendy A, Boersma E (2007). Intensity of statin therapy in relation to myocardial ischemia, troponin T release, and clinical cardiac outcome in patients undergoing major vascular surgery. J Am Coll Cardiol.

[51] Welten GM, Chonchol M, Hoeks SE, Schouten O, Dunkelgrün M, van Gestel YR (2007). Statin therapy is associated with improved outcomes in vascular surgery patients with renal impairment. Am Heart J.

[52] Axelrod DA, Upchurch GR, DeMonner S, Stanley JC, Khuri S, Daley J (2002). Perioperative cardiovascular risk stratification of patients with diabetes who undergo elective major vascular surgery. J Vasc Surg.

[53] Stoner MC, Abbott WM, Wong DR, Hua HT, Lamuraglia GM, Kwolek CJ (2006). Defining the high-risk patient for carotid endarterectomy: an analysis of the prospective National Surgical Quality Improvement Program database. J Vasc Surg.

[54] Halm EA, Tuhrim S, Wang JJ, Rockman C, Riles TS, Chassin MR (2009). Risk factors for perioperative death and stroke after carotid endarterectomy: results of the new york carotid artery surgery study. Stroke.

[55] Fluri F, Hatz F, Voss B, Lyrer PA, Engelter ST (2010). Restenosis after carotid endarterectomy: significance of newly acquired risk factors. Eur J Neurol.

[56] Goodney PP, Nolan BW, Eldrup-Jorgensen J, Likosky DS, Cronenwett JL, Vascular Study Group of Northern New England (2010). Restenosis after carotid endarterectomy in a multicenter regional registry. J Vasc Surg.

