# Supplementary material for: Comparison of outcomes after carotid endarterectomy between type 2 diabetic and non-diabetic patients with significant carotid stenosis
Source: Cardiovasc Diabetol. 2019 Mar 25;18:41. doi: 10.1186/s12933-019-0848-7 (PMC6432752; doi:10.1186/s12933-019-0848-7)
Supplement: Supplementary file 1 — Additional file 1: Table S1. Factors associated with the occurrence of stroke within 30 days after carotid endarterectomy. Table S2. Factors associated with the occurrence of stroke within 4 years after carotid endarterectomy. Table S3. Factors associated with mortality within 4 years after carotid endarterectomy. [file 12933_2019_848_MOESM1_ESM.docx]

**Table S1** Factors associated with the occurrence of stroke within 30 days after carotid endarterectomy

|  | Univariate analysis | | Multivariate analysis | |
| --- | --- | --- | --- | --- |
|  | OR (95% CI) | P-value | OR (95% CI) | P-value |
| Age | 0.99 (0.91–1.08) | 0.774 | NA | NA |
| Female sex | NA | NA | NA | NA |
| BMI | 1.17 (0.94–1.46) | 0.163 | NA | NA |
| Smoking | 1.76 (0.36–8.55) | 0.482 | NA | NA |
| Diabetes | 1.24 (0.33–4.67) | 0.749 | NA | NA |
| Insulin use | NA | NA | NA | NA |
| Hypertension | NA | NA | NA | NA |
| Dyslipidemia | 0.22 (0.05–0.89) | 0.033 | 0.16 (0.04–0.67) | 0.012 |
| CAD | 3.40 (0.90–12.8) | 0.071 | 5.22 (1.30–20.9) | 0.020 |
| Subclinical CAD | NA | NA | NA | NA |
| CKD | 1.44 (0.30–7.04) | 0.649 | NA | NA |
| PAOD | NA | NA | NA | NA |
| Degree of stenosis | 0.99 (0.93–1.06) | 0.814 | NA | NA |
| SCSO | 1.05 (0.13–8.50) | 0.965 | NA | NA |
| Symptomatic stenosis | 0.87 (0.23–3.25) | 0.830 | NA | NA |

BMI, body mass index; CAD, coronary artery disease; CI, confidence interval; CKD, chronic kidney disease; OR, odds ratio; NA, not applicable; PAOD, peripheral arterial occlusive disease; SCSO, severe contralateral extracranial carotid stenosis or occlusion

**Table S2** Factors associated with the occurrence of stroke within 4 years after carotid endarterectomy

|  | Univariate analysis | | Multivariate analysis | |
| --- | --- | --- | --- | --- |
|  | HR (95% CI) | P-value | HR (95% CI) | P-value |
| Age | 1.01 (0.97–1.06) | 0.616 | NA | NA |
| Female sex | 0.79 (0.24–2.60) | 0.697 | 0.81 (0.24–2.67) | 0.723 |
| BMI | 1.03 (0.91–1.17) | 0.645 | NA | NA |
| Smoking | 1.73 (0.74–4.04) | 0.203 | NA | NA |
| Diabetes | 2.81 (1.34–5.90) | 0.006 | 2.55 (1.20–5.41) | 0.015 |
| Insulin use | 0.24 (0.03–1.80) | 0.165 | NA | NA |
| Hypertension | 2.79 (0.85–9.19) | 0.092 | 2.44 (0.74–8.06) | 0.145 |
| Dyslipidemia | 0.68 (0.33–1.42) | 0.304 | NA | NA |
| CAD | 2.34 (1.11–4.91) | 0.025 | 1.99 (0.94–4.22) | 0.074 |
| Subclinical CAD | 0.05 (0.00–230.9) | 0.481 | NA | NA |
| CKD | 0.82 (0.29–2.34) | 0.706 | NA | NA |
| PAOD | 0.47 (0.06–3.45) | 0.457 | 0.37 (0.05–2.69) | 0.323 |
| Degree of stenosis | 1.01 (0.97–1.05) | 0.650 | NA | NA |
| SCSO | 0.90 (0.27–2.97) | 0.862 | NA | NA |
| Symptomatic stenosis | 0.97 (0.47–1.99) | 0.931 | NA | NA |

BMI, body mass index; CAD, coronary artery disease; CI, confidence interval; CKD, chronic kidney disease; HR, hazard ratio; NA, not applicable; PAOD, peripheral arterial occlusive disease; SCSO, severe contralateral extracranial carotid stenosis or occlusion

**Table S3** Factors associated with mortality within 4 years after carotid endarterectomy

|  | Univariate analysis | | Multivariate analysis | |
| --- | --- | --- | --- | --- |
|  | HR (95% CI) | P-value | HR (95% CI) | P-value |
| Age | 1.07 (1.04–1.13) | <0.001 | 1.06 (1.01–1.12) | 0.023 |
| Female sex | 1.26 (0.62–2.56) | 0.524 | 1.32 (0.48–3.60) | 0.587 |
| BMI | 0.89 (0.81–0.98) | 0.016 | 0.93 (0.80–1.07) | 0.296 |
| Smoking | 1.25 (0.71–2.20) | 0.436 | NA | NA |
| Diabetes | 1.13 (0.68–1.90) | 0.636 | NA | NA |
| Insulin use | 2.69 (1.19–6.08) | 0.018 | 2.34 (1.02–5.37) | 0.045 |
| Hypertension | 0.48 (0.29–0.81) | 0.006 | 0.72 (0.27–1.89) | 0.503 |
| Dyslipidemia | 0.86 (0.50–1.48) | 0.591 | NA | NA |
| CAD | 0.94 (0.49–1.81) | 0.853 | NA | NA |
| Subclinical CAD | 0.49 (0.07–3.50) | 0.474 | NA | NA |
| CKD | 2.30 (1.32–4.00) | 0.003 | 1.42 (0.59–3.43) | 0.439 |
| PAOD | 1.00 (0.36–2.77) | 0.996 | 0.95 (0.27–3.38) | 0.932 |
| Degree of stenosis | 1.01 (0.98–1.04) | 0.440 | NA | NA |
| SCSO | 1.15 (0.52–2.53) | 0.729 | NA | NA |
| Symptomatic stenosis | 0.85 (0.51–1.42) | 0.529 | NA | NA |

BMI, body mass index; CAD, coronary artery disease; CI, confidence interval; CKD, chronic kidney disease; HR, hazard ratio; NA, not applicable; PAOD, peripheral arterial occlusive disease; SCSO, severe contralateral extracranial carotid stenosis or occlusion
